# Preparing for a pandemic: highlighting themes for research funding and practice—perspectives from the Global Research Collaboration for Infectious Disease Preparedness (GloPID-R)

**DOI:** 10.1186/s12916-020-01755-y

**Published:** 2020-09-08

**Authors:** Alice Norton, Louise Sigfrid, Adeniyi Aderoba, Naima Nasir, Peter G. Bannister, Shelui Collinson, James Lee, Geneviève Boily-Larouche, Josephine P. Golding, Evelyn Depoortere, Gail Carson, Barbara Kerstiëns, Yazdan Yazdanpanah

**Affiliations:** 1 GloPID-R Secretariat, University of Oxford, Oxford, UK; 2 UK Collaborative on Development Research, London, UK; 3 Centre for Tropical Medicine and Global Health, Nuffield Department of Medicine, University of Oxford, Oxford, UK; 4 University of Medical Sciences Teaching Hospital, Ondo, Ondo State Nigeria; 5 APIN-Supported HIV Treatment Centre, Jos University Teaching Hospital, Jos, Nigeria; 6 Brighton and Sussex Medical School, Brighton, UK; 7 Clinical Research Department, London School of Hygiene and Tropical Medicine, London, UK; 8 Institute of Infection and Immunity Canadian Institutes of Health Research (CIHR), Government of Canada, Ottawa, Canada; 9 Wellcome, London, UK; 10 European Commissions’ Directorate-General for Research & Innovation, European Commission, Brussels, Belgium; 11 INSERM, Paris, France

**Keywords:** Pandemic, Epidemic, Preparedness, COVID-19, Cohorts, Clinical trials, Collaboration

## Background

Funders and researchers around the world are responding to the COVID-19 pandemic at urgent speed, with greater effectiveness and collaboration than ever before. In the past 8 months, the global health research community has collectively generated and shared a huge amount of knowledge in particular into the clinical characterisation, behavioural insights, genetics, epidemiology, viral pathogenesis, clinical management and diagnosis of COVID-19. This is built on substantial prior preparation, with researchers, public health professionals, funders and multilateral bodies in this field having anticipated and prepared for a pandemic for many years. Further knowledge is needed however to control this pandemic and for safe easing of public health measures.

The Global Research Collaboration for Infectious Disease Preparedness (GloPID-R) is an international network of global health funders and stakeholders formed in 2013 to ensure preparedness for a coordinated research response to epidemics and pandemics [1]. GloPID-R aims to address challenges to effective research in epidemics and pandemics, through both preparedness and response activities.

In December 2019, as part of its preparedness activities, GloPID-R convened a Frontiers meeting with their funded clinical trial networks and cohorts along with key stakeholders involved in emerging epidemic and pandemic preparedness and response globally. The aim was to identify how these groups might collaborate in delivering a coordinated research response in the event of an epidemic or pandemic. Now that we are in the midst of the COVID-19 pandemic, it is important to highlight and reflect on the recommendations identified by these participants, to inform the ongoing research funding and practice during the COVID-19 pandemic as well as preparedness for future outbreaks.

## Preparedness themes for research funding and practice

### Research cohorts are valuable tools for building pandemic research responses

Active cohort studies have the potential to play a key role in emerging epidemic and pandemic research. Longitudinal cohorts generate a wealth of data from individual participants about clinical and laboratory outcomes, which allow for a better understanding of effect modifiers such as genetic factors, chronic disease, socio-demographic factors and long-term outcomes than is possible from other study designs. Established cohorts can also function as a broker between emerging disease researchers and the community addressing challenges to the acceptance of research [2].

There was a call for newly funded cohorts to be designed to be both usable and re-usable in the event of new emerging research questions.

### Research capacity and activity mapping are essential to facilitate collaboration and improve targeting of resources

Improved mapping of both global research capacity and ongoing global research activities was identified as necessary to improve identification of opportunities for collaboration and ‘pivoting’ or ‘supplementing’ of ongoing research efforts in outbreaks and improve coordination as pandemics shift globally.

### Research collaboration especially between clinical trial networks and cohorts is essential to improve research outcomes

Coordination, in particular across clinical trials and cohorts, is needed to make the most effective use of scarce resources to ensure that studies are not underpowered due to changes in infection rates in differing geographical areas.

### Sustainability of funding and research capacity during inter-epidemic periods is key to ensure quality research can be initiated rapidly for epidemics and pandemics

Setting up completely new studies during epidemics and pandemics takes substantial time from the funding commitment, developing necessary infrastructure, research processes and approvals and most importantly trust within the community and leads to fragmentation. Therefore, it may be more efficient to build on large existing studies with baseline continuous research activities, which allow the recruitment of patients from the outset of an outbreak.

Strengthening local research capacity and working closely with governments, local and regional partners and communities to develop and lead national research plans are necessary to ensure critical activities.

### Rapid research and funding systems and rapid data sharing are needed to facilitate knowledge generation to improve practice within epidemics and pandemics

Rapid mobilisation of research funds and resources, early engagement with ethics committees and staged approved ethical protocols, adaptive studies and trial designs were all identified as necessary steps to reduce the significant prior delays in initiating research activities in the epidemic response. Funders acknowledged that for many, current funding structures are often not flexible enough to allow quick pivoting or redirection of resources.

Rapid data sharing is needed to accelerate health benefits and outcomes, to facilitate timely dissemination of data to the public for action, and to prevent misinformation. The GloPID-R Data Sharing Roadmap [3] highlights the key steps to address to enable global data sharing, and the meeting highlighted the need to share emerging barriers and potential solutions in its implementation.

### Ethics and social science need to be core to broader epidemic pandemic and research response activities

Ethics should be at the heart of decision-making and an opportunity for researchers to ensure that the optimal value is being obtained from the research for all stakeholders involved, including communities and individuals. Solutions to improve acceptance and uptake of research by healthcare workers and participants are also crucial along with the need for greater inclusion and translation to the practice of qualitative and social sciences studies in epidemics.

## Discussion

These six preparedness recommendations have already been mirrored and in many cases directly informed practice during the COVID-19 research response (see Table 1).

**Table 1:** COVID-19 relevant practice and ongoing priorities linked to the GloPID-R Frontiers meeting recommendations

	GloPID-R Frontiers meeting recommendations	COVID-19-relevant practice and ongoing priorities
1.	**Research cohorts** are valuable tools for building pandemic research responses.	Several cohorts including UK Biobank and a DHSS in Mozambique have already pivoted or enhanced for COVID-19. Further consideration needs to be given by funders and researchers to relevant cohorts for COVID-19 research. Newly created cohorts are being funded and need to be designed in a way in which they can evolve to address future research questions.
2.	**Research capacity and activity mapping** are essential to facilitate collaboration and improve targeting of resources.	For COVID-19, GloPID-R has collaborated with the UK Collaborative on Development Research to strengthen research mapping through the ‘COVID-19 Research Project Tracker’ [4], a live database of funded research projects on COVID-19 mapped to the WHO Research Roadmap for COVID-19. Several other research trackers have been established focusing on clinical research.
3.	**Research collaboration** especially between clinical trial networks and cohorts are essential to improve research outcomes.	Collaboration between cohorts and clinical trial networks is already evident through initiatives such as PREPARE, ALERRT, Pandora-ID-NET, ISARIC and other networks and was further facilitated by networking at the meeting, much of which is now enabling rapid research in the COVID-19 pandemic.
4.	**Sustainability** of funding and research capacity during inter-epidemic periods is key to ensure quality research can be initiated rapidly for epidemics and pandemics.	The COVID-19 pandemic has already shown the benefits of pre-established studies, coordination of study prioritisation and active studies, ready to recruit at the outset of an outbreak. This was the case for the RECOVERY trial and CoCIN cohort in the UK.
5.	**Rapid research and research funding systems** and **rapid data sharing** are needed to facilitate knowledge generation to improve practice within epidemics and pandemics.	The COVID-19 pandemic has resulted in greater rapid data sharing than seen before, enabling accelerated knowledge, but also highlighting the potential risks from the multitude of non-reviewed papers. This makes the GloPID-R data sharing principles of ethical, accessible, transparent, equitable, fair and quality [5] important to highlight again to guide ongoing activities and for funders to implement the GloPID-R data sharing roadmap recommendations to improve processes.
6.	**Ethics and social science** need to be core to broader epidemic pandemic and research response activities.	For COVID-19, we have certainly seen greater inclusion of ethics and social science than in any previous epidemic, and indeed, these have formed two of the priorities for the WHO ‘Coordinated Global Research Roadmap for COVID-19’ [6]. Research is needed to evaluate the implementation of social science research into practice, building the bridge between science and implementation through moving away from traditional silo working towards integrated, multidisciplinary practice, including social scientists, health promotion, public health and clinical practitioners.

There is potential for further leveraging and global coordination of both existing cohorts and clinical trial networks to improve research quality and outcomes during epidemics and pandemics.

Timely, effective epidemic research to improve health outcomes can only be achieved if multidisciplinary research structures, regulatory functions, funding, partnerships and trust are built and maintained sustainably during inter-epidemic periods. Building sustainable research capacity and capability globally needs to be central to research on the COVID-19 pandemic and for future epidemics and pandemics. Sustainable active studies and multidisciplinary networks, with pre-approved protocols positioned strategically globally, need to build upon this.

## Conclusions

Lessons learned from the COVID-19 research response need to be incorporated into a multidisciplinary framework to facilitate rapid, coordinated research funding and support structures for researchers, to provide an even faster and coordinated research response, avoiding redundancy. New funder principles for research in epidemics provide the first step toward this [7].

## Data Availability

Not applicable
